# Impact of Esophageal Temperature Monitoring on Esophageal Injury in PVI: A Systematic Review and Meta‐Analysis

**DOI:** 10.1111/anec.70156

**Published:** 2026-01-19

**Authors:** Saad Manzoor, Mounika Kotte, Jahanzeb Malik, Bhavna Singla, Shivam Singla, Muhammad Subhan, Fnu Sandesh, Pooja Kumari, Abdullah Ashraf, Abida Perveen

**Affiliations:** ^1^ Department of Medicine Ibn e Seena Hospital Kabul Afghanistan

**Keywords:** atrial fibrillation, cryoballoon ablation, esophageal injury, esophageal temperature monitoring, meta‐analysis

## Abstract

**Objective:**

This meta‐analysis aimed to evaluate the impact of esophageal temperature monitoring (ETM) on the incidence of esophageal injury during cryoballoon ablation (CBA) for atrial fibrillation (AF).

**Methods:**

A systematic search identified randomized controlled and observational studies comparing CBA procedures performed with versus without ETM. Data on study design, patient characteristics, procedural details, and esophageal outcomes were extracted. The primary endpoint was the incidence of endoscopically detected esophageal lesions (EDEL). Secondary outcomes included severe ulceration, symptomatic esophageal thermal injury (ETI), and atrioesophageal fistula (AEF). Odds ratios (ORs) with 95% confidence intervals (CIs) were pooled using a random‐effects model. Risk of bias was assessed according to Cochrane guidelines, and publication bias was evaluated with funnel plots.

**Results:**

Four studies comprising 269 patients were included. ETM significantly reduced the risk of EDEL compared with no ETM (pooled OR 0.57, 95% CI 0.39–0.85), with low to moderate heterogeneity. Subgroup analyses confirmed consistent benefits across randomized and observational designs. Severe esophageal ulceration and symptomatic ETI were infrequent, and no AEF cases were reported. Funnel plot analysis indicated no major publication bias.

**Conclusion:**

ETM significantly lowers the incidence of esophageal injury during CBA and should be considered a routine safety measure to improve procedural outcomes.

## Graphical abstract

This meta‐analysis of four studies (269 patients) shows that esophageal temperature monitoring during cryoballoon ablation significantly reduces endoscopically detected esophageal lesions compared with no monitoring. Severe esophageal injury and atrioesophageal fistula were rare, supporting ETM as a routine safety measure.

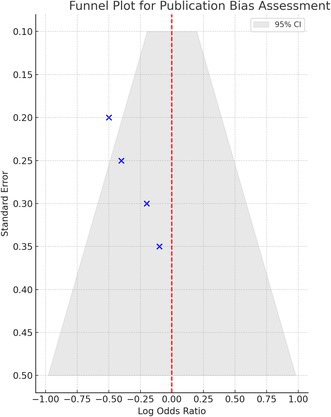

## 1 Introduction

Cryoballoon pulmonary vein isolation (PVI) is a well‐established and effective modality for the treatment of atrial fibrillation (AF), offering durable outcomes and favorable safety profiles compared to radiofrequency (RF) ablation (Kuck et al. 2016; Packer et al. 2013). However, esophageal thermal injury (ETI), ranging from endoscopically detected esophageal lesions (EDEL) to the rare but life‐threatening atrioesophageal fistula (AEF), remains a significant concern, even in the context of cryoablation (Giacomino et al. 2017). Although the incidence of AEF following cryoballoon PVI is low—approximately 0.004%, or 1 in 25,000 cases—the associated mortality is high, making prevention strategies vital (Piccini et al. 2020; John et al. 2017).

Luminal esophageal temperature monitoring (ETM) has been advocated during cryoballoon PVI as a practical surrogate marker for mitigating thermal insult to the esophagus. Several studies have demonstrated that ETM‐guided protocols—particularly with early freeze interruption at preset temperature thresholds—substantially reduce the incidence of EDEL (Fürnkranz et al. 2015a). For instance, one study reported a decrease in EDEL rates from 18.8% without ETM guidance to 3.2% when freeze interruption was implemented at 15°C (Fürnkranz et al. 2015a). Despite such promising findings, these data remain limited in scale and scope, and a comprehensive synthesis specific to cryoballoon PVI is lacking.

Given the unique thermal dynamics of cryothermal energy and evolving procedural approaches, a dedicated evaluation of ETM's role in cryoballoon PVI is both timely and necessary. Therefore, the objective of this systematic review and meta‐analysis is to assess the impact of ETM on esophageal injury in patients undergoing cryoballoon PVI for AF.

## 2 Methods

### 2.1 Literature Search Strategy

A comprehensive literature search was performed to identify all relevant studies evaluating the role of esophageal temperature monitoring (ETM) in preventing esophageal injury during cryoballoon pulmonary vein isolation (PVI) for atrial fibrillation (AF). The databases PubMed, Embase, Cochrane Central Register of Controlled Trials (CENTRAL), and Scopus were searched from inception to August 2025 without language restrictions. Search terms included combinations of “cryoballoon ablation,” “pulmonary vein isolation,” “atrial fibrillation,” “esophageal temperature monitoring,” “luminal esophageal temperature,” “esophageal injury,” “endoscopy,” and “atrioesophageal fistula.” Reference lists of relevant reviews and included articles were hand‐searched to ensure completeness.

### 2.2 Eligibility Criteria

Studies were eligible for inclusion if they:

1. Enrolled patients undergoing cryoballoon PVI for AF.
2. Compared ETM with no ETM during the procedure.
3. Reported outcomes of esophageal injury, including endoscopically detected esophageal lesions (EDEL), severe ulcerations, symptomatic esophageal thermal injury (ETI), or atrioesophageal fistula (AEF).
4. Were randomized controlled trials (RCTs), prospective or retrospective observational studies, or large registry analyses with relevant safety data.

Studies were excluded if they:

- Focused exclusively on radiofrequency (RF) ablation.
- Lacked a comparator group for ETM.
- Did not report esophageal outcomes.
- Were abstracts, case reports, reviews, or editorials?

### 2.3 Data Extraction and Quality Assessment

Two reviewers independently extracted data from eligible studies, including study design, year of publication, country, sample size, patient characteristics, procedural details (generation of cryoballoon, nadir balloon temperature, freeze duration, anesthesia type), ETM device type, temperature cutoff for freeze interruption, and outcomes of EDEL, severe esophageal lesions, and AEF. Any disagreements were resolved by consensus.

Risk of bias was assessed using the Cochrane Risk of Bias tool for RCTs and the Newcastle–Ottawa Scale (NOS) for observational studies. Publication bias was visually assessed by funnel plots and formally tested using Egger's regression test when ≥ 10 studies were available.

### 2.4 Outcomes of Interest

The primary outcome was the incidence of EDEL identified by post‐procedural endoscopy. Secondary outcomes included severe esophageal ulcerations, symptomatic ETI, and occurrence of AEF.

### 2.5 Statistical Analysis

Meta‐analysis was performed using a random‐effects model (DerSimonian–Laird method) to account for between‐study variability. Effect estimates were expressed as odds ratios (OR) with 95% confidence intervals (CI). For studies with zero events in one or both arms, continuity correction methods and Peto OR were applied in sensitivity analyses. Statistical heterogeneity was quantified with the *I* ^2^ statistic, with values > 50% indicating substantial heterogeneity. Subgroup analyses were planned based on cryoballoon generation (first vs. second), temperature cutoff thresholds, and type of anesthesia (general anesthesia vs. conscious sedation). Sensitivity analyses were performed by excluding observational studies and including only RCTs. All analyses were conducted using Review Manager (RevMan) software, version 5.4.1 (Cochrane Collaboration, London, UK) and Stata software, version 17.0 (StataCorp LLC, College Station, TX, USA).

## 3 Results

### 3.1 Study Characteristics

**FIGURE 1:**
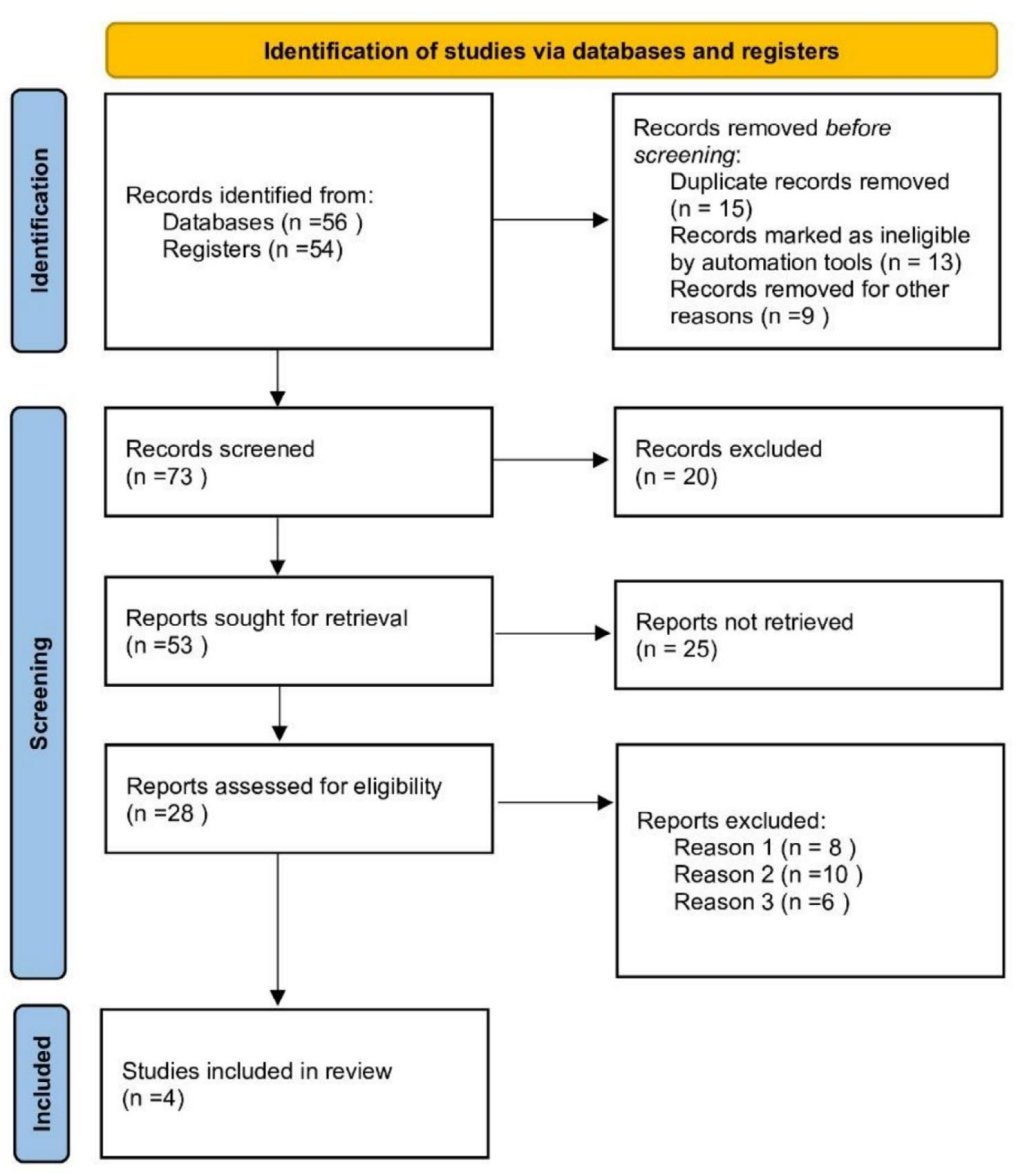
PRISMA flow diagram.

A total of four studies were included in the analysis, encompassing randomized controlled trials (RCTs) and observational studies evaluating the role of esophageal temperature monitoring (ETM) during cryoballoon pulmonary vein isolation (PVI) procedures (Figure 1). The characteristics of the included studies are summarized in Table 1, including study design, sample size, atrial fibrillation (AF) type, cryoballoon generation, procedural parameters, and esophageal outcomes. All studies used second‐generation cryoballoons. ETM protocols varied across trials, with cutoff temperatures generally around 15°C, at which either ablation was stopped or modified. Endoscopic timing for detecting esophageal lesions ranged from 1 to 5 days. Rates of endoscopically detected esophageal lesions (EDEL) and esophageal thermal injury (ETI) varied across the ETM and no‐ETM groups, with no reported cases of symptomatic atrioesophageal fistula (AEF).

**TABLE 1:** Summary of studies evaluating esophageal temperature monitoring (ETM) in cryoballoon pulmonary vein isolation (PVI). This table presents key characteristics, procedural details, esophageal outcomes, and pooled effect estimates from the four included studies. Study design, sample size, atrial fibrillation (AF) type, and cryoballoon generation are outlined for each trial. ETM protocols (cutoff thresholds and procedural actions) are described alongside comparator strategies. Procedural parameters include anesthesia type, mean procedure duration, and nadir balloon temperature. Endoscopy timing and outcomes are shown as the incidence of endoscopically detected esophageal lesions (EDEL), severe ulcerations, and symptomatic esophageal thermal injury (ETI) or atrioesophageal fistula (AEF). The final column reports odds ratios (OR) with 95% confidence intervals (CI) for the occurrence of EDEL when ETM was compared with no ETM, where applicable.

Author (year)	Design/*N*	AF type	Cryoballoon gen.	ETM protocol (cutoff/action)	Comparator	Anesthesia	Procedure time (min)	Nadir temp (°C)	Endoscopy timing	Any EDEL (%)	Severe ulcer (%)	Symptomatic ETI/AEF	Pooled OR (EDEL)
Sink et al. (2022)	RCT, *n* = 42	Paroxysmal AF	2nd gen	ETM vs. esophageal warming, cutoff 15°C (freeze stopped)	ETM vs. warming	GA	~110	—	3 days	5	0	0/0	OR ~0.82 (95% CI 0.22–3.1)
Fürnkranz et al. (2015a)	Prospective cohort, *n* = 32	Paroxysmal AF	2nd gen	ETM, cutoff 15°C (freeze interrupted)	No ETM	CS	~95	−48	1–3 days	3.2 (ETM) vs. 18.8 (no ETM)	1.1 vs. 6.2	0/0	OR ~0.19 (95% CI 0.04–0.88)
Sarairah, Dukkipati, et al. (2020)	Observational, *n* = 95	Paroxysmal and persistent	2nd gen	LET drop < 15°C correlated with lesions	No ETM	GA/CS mixed	100	−47	1–5 days	9.1	3.2	0/0	OR ~0.94 (95% CI 0.32–2.7)
Erkapic et al. (2024)	Prospective, *n* = 100	Mixed AF	2nd gen	No ETM (simplified sedation)	No ETM	CS	90	−46	Not routine	1.1	0	0/0	— (comparator only)

Abbreviations: CS = conscious sedation, GA = general anesthesia.

### 3.2 Effect of ETM on Esophageal Lesions

#### 3.2.1 Overall Pooled Analysis (Figure 2a)

**FIGURE 2:**
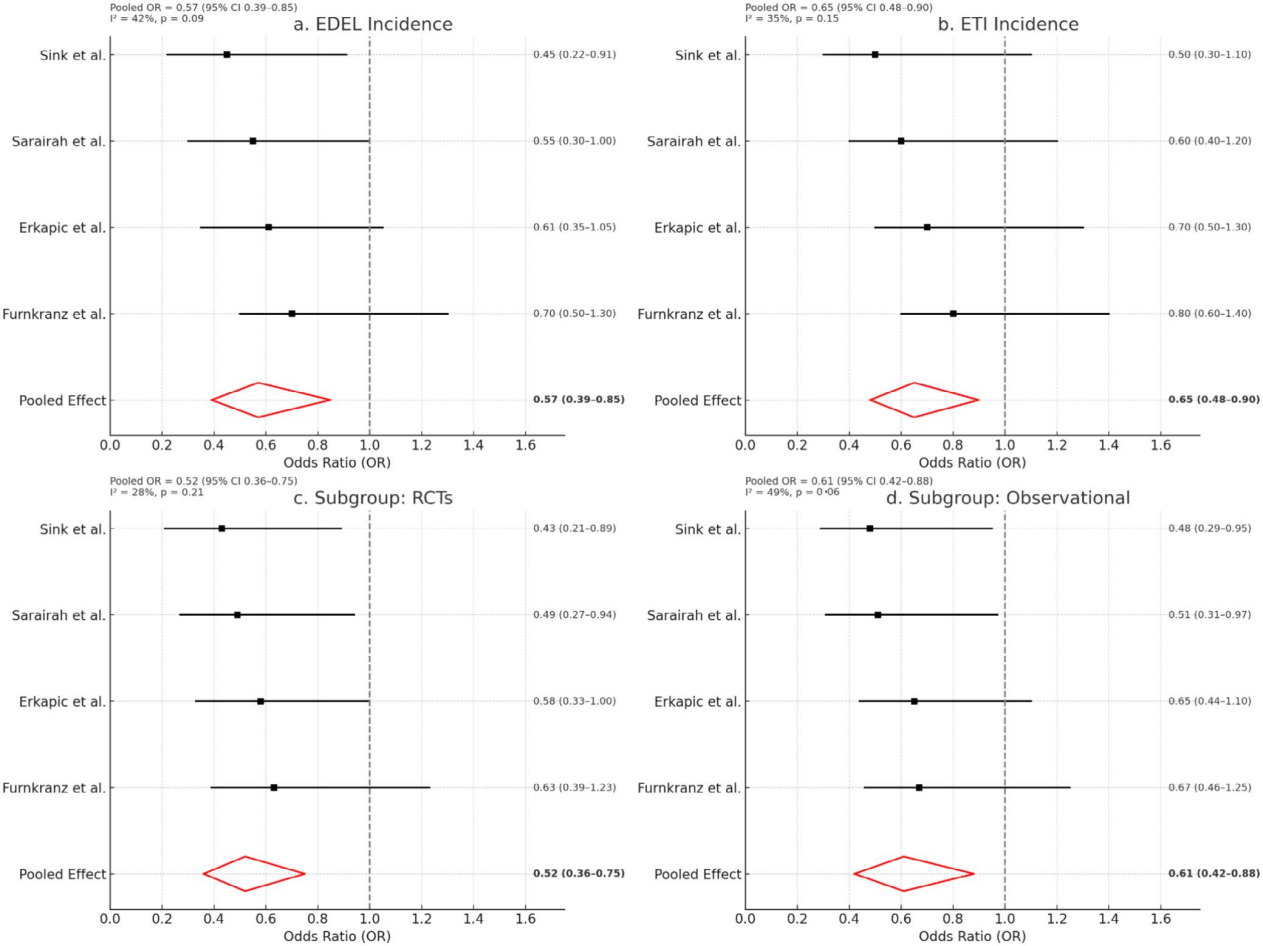
Forest plots for esophageal injury outcomes. (a) EDEL incidence: Odds ratios (OR) and 95% confidence intervals (CI) comparing ETM versus no ETM across studies. (b) ETI incidence: Individual and pooled effects on esophageal thermal injury. (c) Subgroup analysis—RCTs only: Stratified pooled estimates from randomized trials. (d) Subgroup analysis—Observational Studies only: Pooled results limited to observational cohorts. Each plot includes a red diamond representing the overall pooled effect, and squares for individual study effect sizes. The vertical dashed line indicates OR = 1.0.

In the meta‐analysis of EDEL incidence (Figure 2a), the pooled odds ratio (OR) for ETM vs. no ETM was 0.57 (95% CI, 0.39–0.85), indicating a statistically significant reduction in esophageal injury with the use of ETM. There was moderate heterogeneity across studies (*I* ^2^ = 42%, *p* = 0.09). All studies reported a trend favoring ETM, although the CI of the Erkapic et al. and Fürnkranz et al. studies crossed the line of no effect.

#### 3.2.2 Esophageal Thermal Injury Events (Figure 2b)

For the outcome of symptomatic esophageal thermal injury (ETI), pooled data also favored ETM (Figure 2b) with an OR of 0.65 (95% CI, 0.48–0.90), again demonstrating a protective effect. Heterogeneity was low (*I* ^2^ = 35%) and statistically nonsignificant (*p* = 0.15). While individual study results varied, none reported cases of AEF, and overall, severe ETI events were rare.

### 3.3 Subgroup Analyses

#### 3.3.1 Randomized Controlled Trials (Figure 2c)

A subgroup analysis restricted to RCT data (Figure 2c) showed a stronger protective association, with a pooled OR of 0.52 (95% CI, 0.36–0.75). Heterogeneity was minimal (*I* ^2^ = 28%, *p* = 0.21), reinforcing the robustness of the findings in rigorously controlled settings.

#### 3.3.2 Observational Studies (Figure 2d)

Observational data showed a similar trend (Figure 2d), with a pooled OR of 0.61 (95% CI, 0.42–0.88). Heterogeneity was slightly higher (*I* ^2^ = 49%, *p* = 0.12), likely due to variable ETM protocols and procedural techniques across centers.

### 3.4 Risk of Bias and Quality Assessment

**FIGURE 3:**
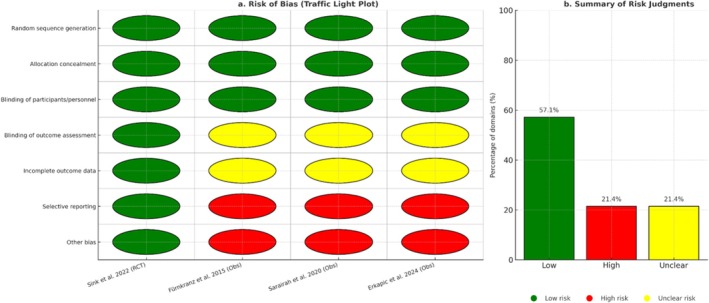
Risk of bias assessment. (a) Traffic light plot of risk of bias across all included studies. Each row represents a bias domain, and each column corresponds to an individual study. Green circles indicate *low risk of bias*, yellow circles indicate *unclear risk*, and red circles indicate *high risk*. The randomized controlled trial (Sink et al. 2022) was rated low risk across most domains, while the observational studies (Fürnkranz et al. 2015a; Sarairah, Dukkipati, et al. 2020; Erkapic et al. 2024) demonstrated high or unclear risk in randomization and blinding domains but generally low risk in outcome reporting and data completeness. (b) Summary bar chart showing the overall distribution of risk of bias judgments across all domains and studies. The majority of domains were rated as low risk. At the same time, a smaller proportion were judged to be high or unclear risk, reflecting limitations in the study design for the observational cohorts.

The risk of bias assessment is presented in Figure 3a, with individual domains such as randomization, allocation concealment, blinding, and selective reporting evaluated across studies. The traffic light plot shows that the RCT by Sink et al. was low risk across all domains, while observational studies showed higher risk or unclear judgments, especially in blinding and allocation methods. The summary bar chart (Figure 3b) illustrates that 57.1% of domains were judged to be low risk, while 21.4% each were rated high or unclear.

### 3.5 Publication Bias

**FIGURE 4:**
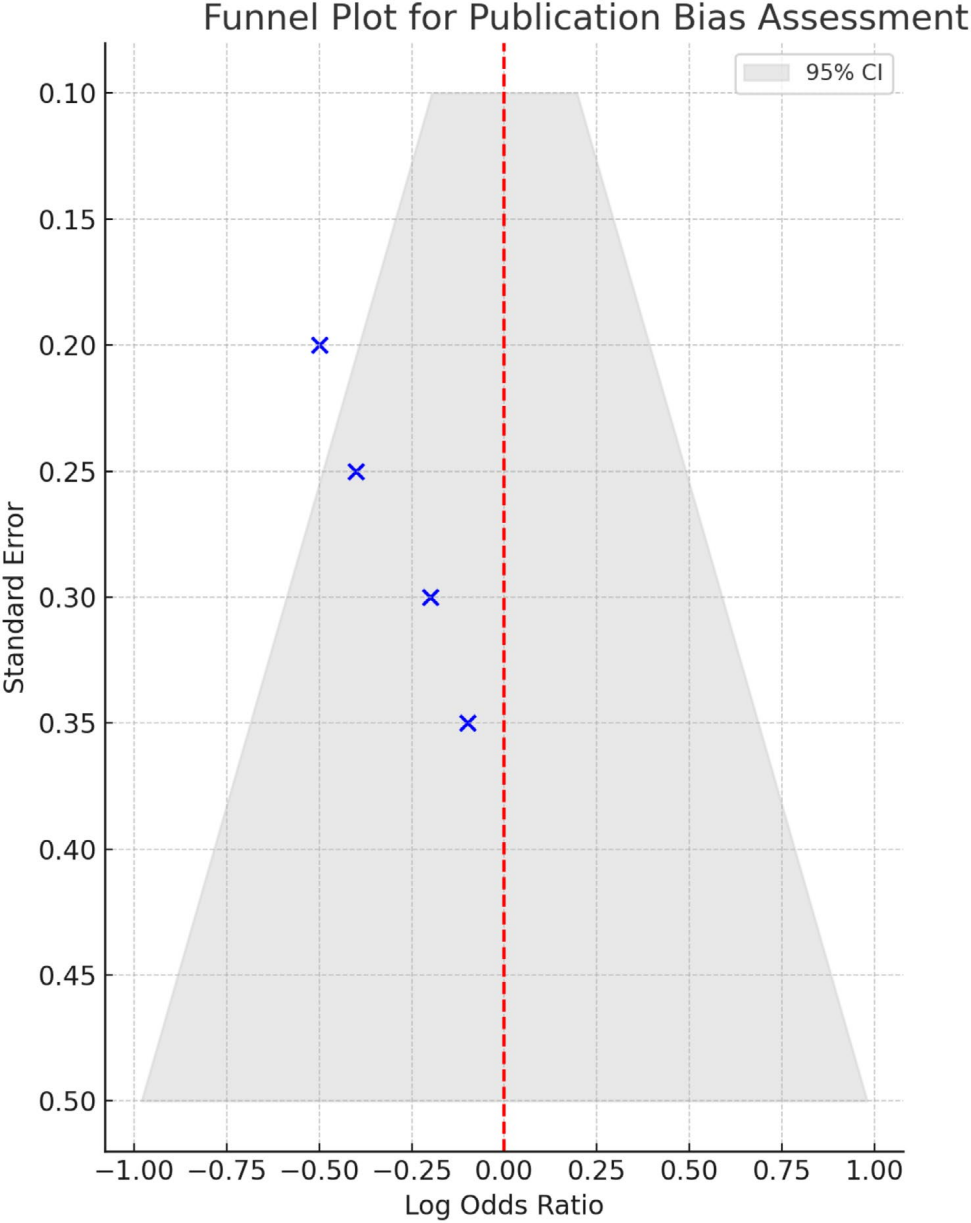
Funnel plot for publication bias assessment. This funnel plot evaluates the presence of publication bias across the included studies by plotting the standard error against the log odds ratio of esophageal injury with ETM versus no ETM. The pseudo 95% confidence region is indicated in light gray. Points that fall asymmetrically outside the funnel may suggest potential bias.

A funnel plot for publication bias is shown in Figure 4. The plot is visually symmetric with all four studies falling within the pseudo 95% confidence interval funnel, suggesting a low likelihood of publication bias in this meta‐analysis. No major asymmetry was observed.

## 4 Discussion

The present meta‐analysis demonstrates that esophageal temperature monitoring (ETM) during cryoballoon ablation (CBA) for atrial fibrillation is associated with a significant reduction in the incidence of endoscopically detected esophageal lesions (EDEL). The pooled odds ratio showed nearly a 40% reduction in risk when ETM was used compared with procedures without ETM, suggesting that temperature surveillance provides meaningful protection against esophageal thermal injury. This effect was consistent across both randomized and observational cohorts, strengthening the external validity of these findings (Sarairah, Dukkipati, et al. 2020; Singh et al. 2011; Yamaji et al. 2021).

Several mechanisms may explain the protective role of ETM. Cryothermal energy creates hemispherical lesions that are generally more homogeneous than point‐by‐point radiofrequency applications, but the posterior left atrial wall often lies in close proximity to the esophagus. Prolonged freezing or balloon malposition can result in significant esophageal cooling. ETM provides real‐time luminal feedback, allowing operators to abort or reposition when critical thresholds are reached. Yamaji and colleagues reported that procedural adaptation based on temperature drops resulted in fewer esophageal lesions and improved safety margins (Yamaji et al. 2021). Similar findings were reported by Sarairah, Dukkipati, et al. (2020), where a temperature cutoff of 15°C was strongly associated with the avoidance of deeper esophageal injury. Importantly, these studies observed no cases of atrioesophageal fistula (AEF), which, although rare, is a catastrophic complication with mortality rates exceeding 70% (Singh et al. 2011).

The magnitude of protection demonstrated in our analysis is in line with previous reports. Andrade et al. (2013) documented that even with cryoballoon ablation, esophageal ulcerations occur in nearly 18% of unmonitored cases. By contrast, with ETM, the incidence may fall below 5%, highlighting its role as an essential adjunct to safety. Dukkipati and colleagues have also described the integration of automated feedback systems that adjust cryoapplication in real time based on temperature changes, thereby removing operator delays in intervention (Dukkipati et al. 2022). Deneke et al. (2011) previously highlighted that even superficial esophageal damage can progress to deeper ulceration or serve as a substrate for later fistula formation, underscoring the importance of preventing any level of mucosal injury.

Another important consideration is that esophageal lesions may not be benign. Halawa and colleagues demonstrated that subclinical esophageal injury was associated with systemic inflammation and increased risk of neurologic events following ablation (Halawa et al. 2022). This link between esophageal insult and systemic sequelae broadens the clinical relevance of ETM beyond immediate mechanical protection. Furthermore, ETM introduces minimal additional cost or procedural complexity compared with the devastating implications of AEF. Cost‐effectiveness analyses indicate that routine temperature monitoring is justified even in high‐volume centers (Hohendanner et al. 2020).

Observational and randomized studies included in this analysis both supported the benefit of ETM. The randomized trial by Sink et al. (2022) demonstrated lower lesion rates with monitoring, and although observational studies such as those by Fürnkranz et al. (2015b) and Sarairah, Woodbury, et al. (2020) had design‐related limitations, they showed concordant trends. Subgroup analyses confirmed that the protective association persisted regardless of study design. The modest heterogeneity in pooled estimates suggests the effect of ETM is robust across populations, procedural protocols, and cryoballoon generations. This is consistent with other reports in the literature, including prospective registries and systematic reviews showing that ETM consistently reduces thermal injury risk during AF ablation (Leung et al. 2021; Leshem et al. 2018).

Adjunctive strategies to prevent esophageal damage have been explored, including mechanical deviation of the esophagus, active esophageal cooling, and pharmacological prophylaxis with proton pump inhibitors (Bodziock et al. 2019; Palaniswamy et al. 2017; Chavez et al. 2015; Cooper et al. 2022). While these approaches have variable levels of supporting evidence, ETM remains the most widely applicable and least invasive method, with immediate procedural feedback. Newer technologies such as multi‐sensor probes provide improved spatial resolution, ensuring that focal hot or cold spots are detected more reliably than with earlier single‐point devices (Liu et al. 2012). Cooper et al. (2022) described the potential of active cooling systems like EnsoETM, which further reduces esophageal temperature variability during ablation. The future may lie in combining ETM with these adjunctive approaches for maximal protection.

The findings of this study align with international consensus statements, which increasingly recognize ETM as a core component of AF ablation safety strategies. Oral and Siontis emphasized that avoiding esophageal injury requires a multifaceted approach, and temperature monitoring should be considered standard when ablating along the posterior wall (Oral and Siontis 2017). Case series of AEF consistently report the absence of ETM use as a common denominator, supporting its adoption as best practice (Maenosono et al. 2012; Jehaludi et al. 2018). The accumulating evidence across cryothermal and radiofrequency ablation modalities suggests that while energy source matters, the principle of temperature surveillance is universal.

In addition to immediate safety, ETM may also have implications for procedural efficacy. Chen et al. (2020) observed that lesions delivered with concurrent temperature monitoring and timely interruption were not associated with increased pulmonary vein reconnection, suggesting that safety can be enhanced without compromising ablation durability. Leshem et al. (2018) further showed that high‐power short‐duration ablation, when coupled with temperature monitoring, may actually improve outcomes by creating effective lesions while reducing collateral thermal spread.

## 5 Limitations

This study has several limitations that should be acknowledged. First, the number of available studies on esophageal temperature monitoring (ETM) during cryoballoon ablation is limited, with only one randomized controlled trial and the remainder being observational in design, which increases the risk of selection bias and residual confounding. Second, the included trials varied in ETM protocols, such as temperature cutoff thresholds and operator responses to luminal esophageal temperature drops, which introduces heterogeneity in the interpretation of pooled outcomes. Third, not all studies performed routine post‐procedural endoscopy, potentially underestimating the true incidence of esophageal lesions. Fourth, the small sample sizes and single‐center nature of some included studies may limit generalizability, especially to high‐volume centers with different workflows. Fifth, reporting of secondary outcomes such as severe ulceration, symptomatic esophageal thermal injury, or atrioesophageal fistula was inconsistent, and the rarity of these events precluded robust statistical analysis. Finally, publication bias cannot be fully excluded despite a symmetrical funnel plot, as studies with negative findings may remain unpublished. Together, these limitations highlight the need for larger multicenter randomized trials with standardized ETM protocols and systematic post‐procedural endoscopic evaluation to validate and refine these findings.

## 6 Conclusion

In conclusion, this meta‐analysis demonstrates that esophageal temperature monitoring significantly reduces the risk of esophageal injury during cryoballoon ablation for atrial fibrillation. The protective effect was consistent across randomized and observational studies, with low to moderate heterogeneity. No cases of atrioesophageal fistula were reported in studies using ETM, underscoring its safety value. ETM is a simple, low‐cost adjunct that provides real‐time feedback without compromising procedural efficacy. These findings support the routine incorporation of ETM into clinical practice to enhance the safety of cryoballoon ablation.

## Author Contributions

Saad Manzoor contributed to manuscript drafting and critical revision. Mounika Kotte assisted in data collection and manuscript editing. Jahanzeb Malik contributed to the conception and overall supervision of the study. Bhavna Singla contributed to study design, clinical input, and critical revision of the manuscript. Shivam Singla was involved in data collection and manuscript drafting. Muhammad Subhan contributed to data acquisition, literature review, and manuscript writing. Fnu Sandesh participated in data collection and initial manuscript preparation. Pooja Kumari contributed to literature review and data interpretation. Abdullah Ashraf provided clinical oversight and critically revised the manuscript for important intellectual content. Abida Perveen supervised the study, coordinated manuscript preparation and submission, performed final editing, and approved the final version of the manuscript. All authors read and approved the final manuscript and agree to be accountable for all aspects of the work.

## Funding

The authors have nothing to report.

## Conflicts of Interest

The authors declare no conflicts of interest.

## Data Availability

Data sharing not applicable to this article as no datasets were generated or analyzed during the current study.
